# High HBV-DNA serum levels are associated with type 2 diabetes in adults with positive HBsAg: An observational study

**DOI:** 10.3389/fendo.2023.1146798

**Published:** 2023-04-03

**Authors:** Sijia Zhang, Yan Zong, Yue Hu, Yuhan Sheng, Guangqin Xiao

**Affiliations:** Cancer Center, Union Hospital, Tongji Medical College, Huazhong University of Science and Technology, Wuhan, China

**Keywords:** type 2 diabetes, hepatitis B virus, HBV-DNA, fasting plasma glucose, glycated hemoglobin

## Abstract

**Background:**

The prevalence of diabetes is higher in hepatitis B virus (HBV)-infected population. We aimed to examine the relationship between different serum HBV-DNA levels and type 2 diabetes in adults with positive HBV surface antigen (HBsAg).

**Methods:**

We conducted cross-sectional analyses of data obtaining from the Clinical Database System of Wuhan Union Hospital. Diabetes was defined by self-report of type 2 diabetes, fasting plasma glucose (FPG) ≥7mmol/L, or glycated hemoglobin (HbA1c) ≥6.5%. Binary logistic regression analyses were performed to investigate the factors associated with diabetes.

**Results:**

Among 12,527 HBsAg-positive adults, 2,144 (17.1%) were diabetic. Patients with serum HBV-DNA <100, 100-2000, 2000-20000 and ≥20000 IU/mL accounted for 42.2% (N=5,285), 22.6% (N=2,826), 13.3% (N=1,665) and 22.0% (N=2,751), respectively. The risk of type 2 diabetes, FPG ≥7mmol/L and HbA1c ≥6.5% in individuals with highly elevated serum HBV-DNA level (≥20000 IU/mL) were 1.38 (95% confidence interval [CI]: 1.16 to 1.65), 1.40 (95% CI: 1.16 to 1.68) and 1.78 (95% CI: 1.31 to 2.42) times relative to those with negative or lowly elevated serum HBV-DNA (<100 IU/mL). However, the analyses showed no association of moderately (2000-20000 IU/mL) to slightly (100-2000 IU/mL) raised serum HBV-DNA levels with type 2 diabetes (OR=0.88, P=0.221; OR=1.08, P=0.323), FPG ≥7mmol/L (OR=1.00, P=0.993; OR=1.11, P=0.250) and HbA1c ≥6.5% (OR=1.24, P=0.239; OR=1.17, P=0.300).

**Conclusion:**

In HBsAg-positive adults, highly elevated level rather than moderately to slightly raised levels of serum HBV-DNA is independently associated with an increased risk of type 2 diabetes.

## 1 Introduction

Diabetes mellitus is a kind of metabolic disease characterized by hyperglycemia (1, 2). At present, approximately 463 million people aged from 20 to 79 years old are suffering from diabetes worldwide, most of whom are type 2 diabetes (3, 4). Long-term hyperglycemia can lead to chronic damage and dysfunction of many organs such as eyes, kidneys, heart, blood vessels and nerves (5–7). Diabetes also can increase the risk of many common cancers, including liver cancer, pancreatic cancer, endometrial cancer, colorectal cancer and breast cancer (8, 9). Studies report that more than ten percent of global all-cause mortality is associated with diabetes (10, 11). Hence, diabetes and premature death and disability caused by diabetes have become a major public health event threatening human life. The incidence of diabetes is related to a variety of risk factors in adult population (12, 13). Studies have shown that multiple viral infections such as Hepatitis B virus (HBV) or Hepatitis C virus (HCV) are independently associated with increased risk of type 2 diabetes in adults (14–16).

HBV infection is one of the most prevalent infectious diseases worldwide. According to estimation, in the global, approximately 248 million people are HBV surface antigen (HBsAg) positive, more than half in sub-Saharan Africa and east Asia (17, 18). There are about 1.4 million people dying per year from acute liver failure, cirrhosis and liver cancer caused by HBV infection worldwide (17, 19). China is a highly endemic area of HBV infection. The estimated total number of people with positive HBsAg has exceeded more than 100 million (20). In patients with HBV infection, serum HBV-DNA level is an important marker of the viral load in the host. And persistent high level of serum HBV-DNA play a pivotal role in cirrhosis pathogenesis and liver cancer carcinogenesis (21, 22). Moreover, studies have shown that elevated serum HBV-DNA level can increase the risk of diabetes in patients with HBV infection (23, 24). However, other studies have suggested that positive serum HBV-DNA is not correlated with the risk of diabetes (25, 26). Hence, the correlation between serum HBV-DNA level and risk of type 2 diabetes is not completely specified in patients with HBV infection.

Therefore, we conducted a cross-sectional observational study in Chinese adults with positive HBsAg based on a large sample from the database system of Wuhan Union hospital. We aimed to investigate the relationship between different serum HBV-DNA levels and type 2 diabetes, fasting plasma glucose (FPG) equal to or greater than 7 mmol/L, or glycosylated hemoglobin (HbA1c) equal to or greater than 6.5%.

## 2 Methods

From January 2014 to January 2023, the Clinical Database System of Wuhan Union Hospital recorded the data of patients hospitalized in Wuhan Union Hospital. We conducted a cross-sectional observational study by analyzing the data extracted from this database system. Patients meeting the following criteria were included: 1. Patients were equal to or older than 18 years old; 2. Patients had positive results of serum HBsAg. The exclusion criteria of this study were: 1. Lack of serum HBV-DNA results; 2. Missing the data of age; 3. Patients without results of FPG and HbA1c. This study was approved by the ethics committee of Wuhan Union Hospital of Huazhong University of Science and Technology. And it was performed in accordance with the ethical standards formulated in the Helsinki Declaration.

The collection of demographic characteristics of inpatients in our hospital was completed by trained doctors. The general information of the patients included age, gender and past medical history. The diagnosis for diabetes was based on the 2021 diabetes diagnostic standard established by the American Diabetes Association (ADA). Namely, the adults had at least one of the following items were diagnosed as having diabetes: with type 2 diabetes reported by oneself, receiving therapy for type 2 diabetes, FPG equal to or greater than 7 mmol/L, HbA1c equal to or greater than 6.5%.

The venous blood of the included patients was collected on the morning of the next day after hospitalization after fasting for at least ten hours, and then be sent for examination immediately. The concentration of red blood cell (RBC), white blood cell (WBC) and hemoglobin were tested by Beckman counter (UniCel DXH 800 counter cellular analyses system or counter LH 750 analyzer). The instruments used for the detection of coagulation function variables (such as prothrombin time [PT], thrombin time [TT], activated partial prothrombin time [APTT], International standardized ratio [INR] and fibrinogen) were automatic coagulation analyzers (Thrombolyzer-XRM, Behnk Elektronik, Germany). Blood biochemical variables (including bilirubin, alanine aminotransferase [ALT], aspartate aminotransferase [AST], alkaline phosphatase [ALP], gamma glutamyl transpeptidase [GGT], cholesterol and FPG) were measured by automatic biochemical analyzers (Abbott ci8200 or BECKMAN COULTER AU5800). The level of HbA1c in blood was detected by D-10 glycated hemoglobin analyzer (Bio-Rad, USA).

In this study, all patients received the test for serum HBsAg, HBV e antigen (HBeAg) and HBV-DNA levels. HBsAg and HBeAg were measured by automatic enzyme linked immunosorbent assay system (FREEDOM EVOLYZER, Tecan, Schweiz). Quantitative real-time polymerase chain reaction was conducted to measure the serum HBV-DNA level (Applied Biosystems 7500, USA). The patients were divided into four groups according to the serum HBV-DNA level: patients with negative or lowly elevated serum HBV-DNA (<100 IU/mL), patients with slightly elevated serum HBV-DNA (≥100, <2000 IU/mL), patients with moderately raised serum HBV-DNA (≥2000, <20000 IU/mL) and patients with highly increased serum HBV-DNA (≥20000 IU/mL). As Hong Li et al. (27) found that HBsAg at a baseline level of 200 IU/mL was more predictive of carriers of HBsAg, so we set this value as the cutoff for HBsAg index.

Statistical Package for Social Sciences (SPSS) version 25 (IBM, Chicago, IL, USA) was used for statistical analyses. Nonparametric Kolmogorov-Smirnov test was applied to all the continuous variables to determine whether the variables conform to the normal distribution. Independent sample t test was conducted to compare the mean values for the continuous variables of normal distribution. Nonparametric test was used to examine the median for the nonnormal distribution variables. Categorical data were analyzed by chi-square or Fisher exact tests. Univariate binary logistic regression analyses were conducted to screen variables associated with total diabetes, elevated FPG and HbA1c with the unadjusted odds ratio (OR) and 95% confidence interval (CI). For variables with a P value less than 0.15 in univariate analyses were selected into binary logistic regression analyses to determine the independent factors associated with total diabetes, elevated FPG and HbA1c with the adjusted odds ratio (OR) and 95% CI. A two-tailed p value less than 0.05 was considered statistically significant.

## 3 Results

**Figure 1:**
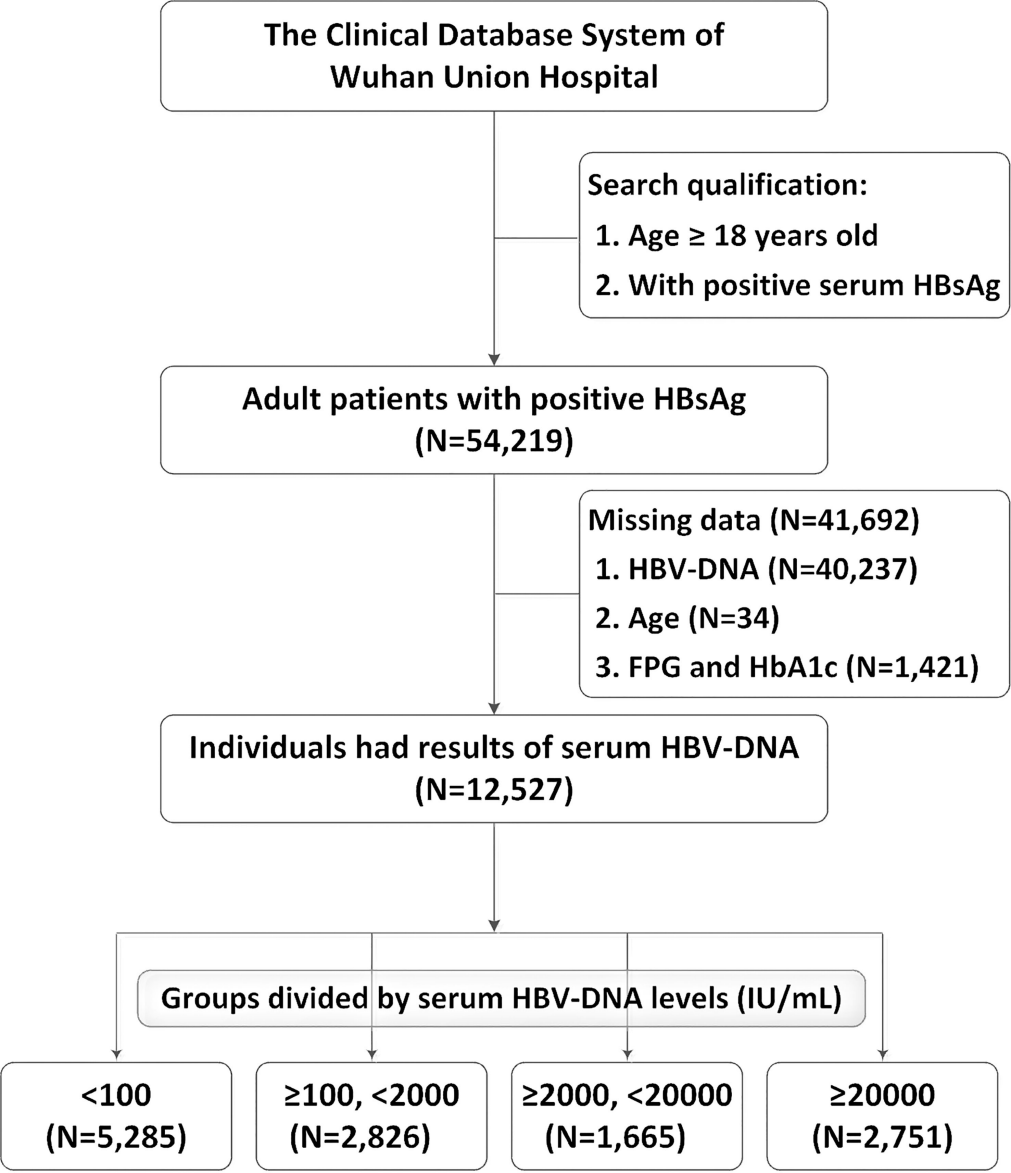
Flow-chart of the study population selection. DNA, deoxyribonucleic acid; HbA1c, glycated hemoglobin; HBsAg, hepatitis B surface antigen; HBV, Hepatitis B Virus; FPG, fasting plasma glucose.

After screening for age and serum HBsAg, the data of 54,219 adults with positive HBsAg were extracted from the Clinical Research Retrieval Database System of Wuhan Union Hospital (From January 2014 to January 2023). 41,692 cases were excluded from this study: 40,237 patients had no results of serum HBV-DNA, 34 patients missed the age data, and 1,421 patients were lacking of results neither FPG nor HbA1c. Finally, 12,527 patients were included for analyses. The flowchart of population selection was showed in **Figure 1**. There were 5,285 (42.2%) patients with negative or lowly elevated serum HBV-DNA (<100 IU/mL). 2,826 (22.6%) and 1,665 (13.3%) patients had slightly (≥100, <2000 IU/mL) or moderately (≥2000, <20000 IU/mL) elevated serum HBV-DNA, respectively. The number of patients with highly increased serum HBV-DNA levels was 2,751 (22.0%). The general characteristics and blood tests of all patients were shown in **Table 1**. Among the included patients, there were 8,722 (69.6%) males and 3,805 (30.4%) females. The mean age of all individuals was 53.2 (standard deviation [SD]: 12.5) years old. The percentage of total type 2 diabetes, FPG ≥7mmol/L, HbA1c ≥6.5% accounted for 17.1% (2,144/12,527), 11.0% (1,383/12,527) and 25.4% (503/1977), respectively.

**Table 1:** General characteristics of HBsAg-positive adults with different levels of serum HBV-DNA.

Characteristics	Overall (N=12,527)	<100 IU/mL (N=5,285)	≥100, <2000 IU/mL (N=2,826)	≥2000, <20000 IU/mL (N=1,665)	≥20000 IU/mL (N=2,751)
Sex					
Male	8,722 (69.6%)	3,604 (68.2%)	1,884 (66.7%)	1,162 (69.8%)	2,072 (75.3%)
Female	3,805 (30.4%)	1,681 (31.8%)	942 (33.3%)	503 (30.2%)	679 (24.7%)
HBsAg					
≥200 (IU/mL)	6,853 (60.6%)	2,076 (44.1%)	1,477 (56.4%)	1,095 (72.2%)	2,205 (89.6%)
<200 (IU/mL)	4,454 (39.4%)	2,635 (55.9%)	1,140 (43.6%)	422 (27.8%)	257 (10.4%)
HBeAg					
Positive	1,633 (13.0%)	285 (5.4%)	151 (5.3%)	160 (9.6%)	1,037 (37.7%)
Negative	10,889 (87.0%)	5,000 (94.6%)	2,674 (94.7%)	1,503 (90.4%)	1,712 (62.3%)
HCVAb					
Positive	177 (1.6%)	93 (2.0%)	41 (1.6%)	19 (1.2%)	24 (1.0%)
Negative	11,168 (98.4%)	4,619 (98.0%)	2,534 (98.4%)	1,517 (98.8%)	2,498 (99.0%)
Total diabetes					
Yes	2,144 (17.1%)	906 (17.1%)	482 (17.1%)	238 (14.3%)	518 (18.8%)
No	10,383 (82.9%)	4,379 (82.9%)	2,344 (82.9%)	1,427 (85.7%)	2,233 (81.2%)
FPG					
≥7mmol/L	1,383 (11.0%)	558 (10.6%)	301 (10.7%)	163 (9.8%)	361 (13.1%)
<7mmol/L	11,144 (89.0%)	4,727 (89.4%)	2,525 (89.3%)	1,502 (90.2%)	2,390 (86.9%)
HbA1c					
≥6.5%	503 (25.4%)	199 (23.5%)	116 (23.2%)	65 (24.4%)	123 (33.7%)
<6.5%	1,474 (74.6%)	648 (76.5%)	383 (76.8%)	201 (75.6%)	242 (66.3%)
Mean Age (Years)	53.2 (12.5)	53.4 (12.5)	53.8 (12.1)	53.5 (11.9)	51.9 (13.1)
FPG (mmol/L)	4.98 (4.50-5.70)	5.00 (4.53-5.72)	4.99 (4.50-5.64)	4.96 (4.50-5.64)	4.89 (4.40-5.70)
HbA1c (%)	5.7 (5.3-6.5)	5.7 (5.3-6.4)	5.7 (5.3-6.3)	5.7 (5.3-6.4)	5.9 (5.3-6.9)
White blood cell (x10^9^/L)	5.38 (4.11-6.97)	5.35 (4.06-6.94)	5.54 (4.27-7.08)	5.48 (4.25-7.00)	5.18 (3.98-6.86)
Red blood cell (x10^12^/L)	4.14 (3.64-4.58)	4.13 (3.60-4.59)	4.18 (3.73-4.59)	4.18 (3.71-4.61)	4.10 (3.57-4.53)
Hemoglobin (g/L)	127.0 (111.0-141.0)	126.0 (108.0-141.0)	127.0 (113.0-141.0)	129.0 (114.0-143.0)	127.0 (111.0-141.0)
Platelet (x10^9^/L)	167.0 (111.0-219.0)	169.0 (110.0-224.0)	176.0 (127.0-228.8)	171.0 (119.0-219.3)	150.0 (96.0-201.0)
APTT (s)	37.6 (34.9-40.8)	37.5 (34.7-40.6)	37.1 (34.6-40.4)	37.3 (35.1-40.4)	38.2 (35.4-41.6)
Prothrombin Time (s)	13.7 (13.0-14.7)	13.5 (12.9-14.5)	13.5 (12.9-14.4)	13.6 (13.0-14.6)	14.1 (13.2-15.4)
INR	1.06 (0.99-1.17)	1.05 (0.98-1.15)	1.05 (0.99-1.14)	1.06 (1.00-1.16)	1.11 (1.02-1.24)
Fibrinogen (g/L)	2.99 (2.41-3.93)	3.06 (2.46-4.04)	3.05 (2.50-3.94)	2.93 (2.38-3.81)	2.86 (2.25-3.77)
Thrombin Time (s)	17.9 (16.8-19.2)	17.7 (16.7-18.9)	17.8 (16.7-19.0)	17.9 (16.8-19.2)	18.5 (17.3-20.0)
Total bilirubin (μmol/L)	13.9 (9.9-20.6)	13.2 (9.4-19.2)	13.5 (9.8-19.2)	13.8 (10.1-20.6)	16.1 (11.1-25.8)
ALT (IU/L)	28.0 (18.0-50.0)	24.0 (16.0-39.0)	25.0 (16.0-42.0)	29.0 (19.0-50.0)	46.0 (29.0-92.0)
AST (IU/L)	28.0 (20.0-51.0)	25.0 (19.0-38.0)	25.0 (19.0-42.0)	28.0 (21.0-50.0)	47.0 (29.0-94.0)
ALP (IU/L)	81.0 (63.0-113.0)	78.0 (62.0-105.0)	77.0 (61.0-107.0)	80.0 (62.0-111.0)	94.0 (70.0-132.0)
GGT (IU/L)	30.0 (17.0-75.0)	27.0 (16.0-57.0)	26.0 (16.0-62.0)	30.0 (16.0-73.0)	51.0 (24.0-120.0)
Total protein (g/L)	64.9 (60.0-70.0)	65.1 (60.0-70.1)	65.4 (60.9-70.2)	64.9 (60.4-69.9)	64.0 (58.8-69.1)
Albumin (g/L)	38.6 (34.1-42.2)	39.0 (34.8-42.7)	39.4 (35.2-42.6)	39.0 (34.8-42.4)	36.4 (31.7-40.6)
Triglycerides (mmol/L)	1.00 (0.74-1.43)	1.02 (0.74-1.49)	1.03 (0.76-1.48)	0.98 (0.75-1.38)	0.95 (0.72-1.29)
Total Cholesterol (mmol/L)	3.92 (3.27-4.65)	3.88 (3.25-4.63)	4.04 (3.40-4.72)	3.99 (3.37-4.68)	3.80 (3.12-4.61)
HDL Cholesterol (mmol/L)	1.09 (0.86-1.36)	1.09 (0.86-1.34)	1.12 (0.89-1.40)	1.12 (0.88-1.39)	1.05 (0.80-1.32)
LDL Cholesterol (mmol/L)	2.28 (1.75-2.87)	2.25 (1.77-2.85)	2.37 (1.82-2.96)	2.35 (1.78-2.92)	2.21 (1.64-2.79)

ALP, Alkaline Phosphatase; ALT, Alanine Aminotransferase; APTT, Activated Partial Thromboplastin Time; AST, Aspartate Aminotransferase; DNA, Deoxyribonucleic Acid; FPG, Fasting Plasma Glucose; GGT, Gamma-glutamyl Transferase; HbA1c, Glycated Hemoglobin; HBeAg, Hepatitis B e Antigen; HBsAg, Hepatitis B Surface Antigen; HBV, Hepatitis B Virus; HCV, Hepatitis C Virus. HDL, High-density Lipoprotein; INR, International Normalized Ratio; IQR, Interquartile Range; LDL, Low-density Lipoprotein; SD, Standard Deviation. Data are n (%), mean (SD) or median (IQR).

### 3.1 Relationship between slightly raised serum HBV-DNA level and type 2 diabetes

As shown in **Table 1**, among 2,826 patients with slight increase of serum HBV-DNA (≥100, <2000 IU/mL) (with mean age of 53.8 years, SD: 12.1 years), 1,884 (66.7%) and 942 (33.3%) were males and females. The percentages of the adults with total diabetes, FPG ≥7mmol/L, HbA1c ≥6.5% were (17.1%) (482/2826), 10.7% (301/2826) and 23.2% (116/499), respectively. Compared to negative or lowly elevated serum HBV-DNA (<100 IU/mL), the unadjusted ORs of the relationship between slightly raised serum HBV-DNA level and total diabetes, FPG ≥7mmol/L and HbA1c ≥6.5% were 0.99 (95% CI: 0.88-1.12, P=0.921), 1.01 (95% CI: 0.87-1.17, P=0.897) and 0.99 (95% CI: 0.76-1.28, P=0.917), respectively. Multivariate binary logistic regression analyses demonstrated that there was no correlation between slightly increased serum HBV-DNA and total diabetes, FPG ≥7mmol/L and HbA1c ≥6.5%, with adjusted ORs of 1.08 (95% CI: 0.93-1.26, P=0.323), 1.11 (95% CI: 0.93-1.33, P=0.250) and 1.17 (95% CI: 0.87-1.56, P=0.300), respectively (As showed in **Tables 2**–**4**).

**Table 2:** Univariate and multivariate binary logistic regression analyses identifying potential factors associated with total type 2 diabetes.

Variables	Unadjusted OR	95% CI	p-Value	Adjusted OR	95% CI	p-Value
Sex: Male	1.26	1.34-1.40	<0.001	1.26	1.09-1.45	0.002
HBsAg: ≥200	0.75	0.68-0.82	<0.001	0.80	0.70-0.91	0.001
HBeAg: Positive	0.74	0.64-0.86	<0.001	0.78	0.63-0.96	0.022
HCVAb: Positive	1.42	0.99-2.03	0.057	–	–	–
HBV-DNA	–	–	0.002	–	–	<0.001
≥20000 IU/mL	1.12	1.00-1.26	0.060	1.38	1.16-1.65	<0.001
≥2000, <20000 IU/mL	0.81	0.69-0.94	0.006	0.88	0.72-1.08	0.221
≥100, <2000 IU/mL	0.99	0.88-1.12	0.921	1.08	0.93-1.26	0.323
Age (Yeas)	1.03	1.02-1.03	<0.001	1.03	1.02-1.03	<0.001
White blood cell (x10^9^/L)	1.01	1.00-1.01	<0.001	1.01	1.00-1.01	0.049
Red blood cell (x10^12^/L)	0.81	0.76-0.85	<0.001	0.85	0.78-0.92	<0.001
Hemoglobin (g/L)	0.99	0.99-1.00	<0.001	–	–	–
Platelet (x10^9^/L)	1.00	1.00-1.00	<0.001	1.00	1.00-1.00	<0.001
APTT (s)	0.98	0.97-0.99	<0.001	0.96	0.95-0.97	<0.001
Prothrombin Time (s)	1.02	1.01-1.03	0.007	1.21	1.05-1.39	0.008
INR	1.13	1.01-1.25	0.030	0.31	0.10-0.93	0.036
Fibrinogen (g/L)	1.09	1.05-1.13	<0.001	1.15	1.09-1.20	<0.001
Thrombin Time (s)	1.00	0.99-1.01	0.487	–	–	–
Total bilirubin (μmol/L)	1.00	1.00-1.00	0.151	–	–	–
ALT (IU/L)	1.00	1.00-1.00	0.004	–	–	–
AST (IU/L)	1.00	1.00-1.00	0.119	–	–	–
ALP (IU/L)	1.00	1.00-1.00	0.624	–	–	–
GGT (IU/L)	1.00	1.00-1.00	0.005	–	–	–
Total protein (g/L)	0.99	0.99-1.00	0.003	–	–	–
Albumin (g/L)	0.97	0.97-0.98	<0.001	–	–	–
Triglycerides (mmol/L)	1.27	1.21-1.33	<0.001	1.30	1.22-1.38	<0.001
Total Cholesterol (mmol/L)	0.94	0.91-0.98	0.004	–	–	–
HDL Cholesterol (mmol/L)	0.50	0.44-0.56	<0.001	0.65	0.55-0.77	<0.001
LDL Cholesterol (mmol/L)	0.93	0.88-0.98	0.005	–	–	–

ALP, Alkaline Phosphatase; ALT, Alanine Aminotransferase; APTT, Activated Partial Thromboplastin Time; AST, Aspartate Aminotransferase; CI, Confidence Interval; DNA, Deoxyribonucleic Acid; GGT, Gamma-glutamyl Transferase; HBeAg, Hepatitis B e Antigen; HBsAg, Hepatitis B Surface Antigen; HBV, Hepatitis B Virus; HCV, Hepatitis C Virus. HDL, High-density Lipoprotein; INR, International Normalized Ratio; LDL, Low-density Lipoprotein; OR, Odds Ratio.

**Table 3:** Univariate and multivariate binary logistic regression analyses investigating potential factors associated with FPG ≥7mmol/L.

Variables	Unadjusted OR	95% CI	p-Value	Adjusted OR	95% CI	p-Value
Sex: Male	1.43	1.26-1.63	<0.001	1.34	1.13-1.58	0.001
HBsAg: ≥200	0.88	0.79-0.99	0.039	–	–	–
HBeAg: Positive	0.87	0.73-1.04	0.123	0.76	0.60-0.96	0.023
HCVAb: Positive	1.21	0.78-1.88	0.403	–	–	–
HBV-DNA	–	–	0.001	–	–	0.004
≥20000 IU/mL	1.28	1.11-1.47	0.001	1.40	1.16-1.68	<0.001
≥2000, <20000 IU/mL	0.92	0.76-1.10	0.370	1.00	0.80-1.25	0.993
≥100, <2000 IU/mL	1.01	0.87-1.17	0.897	1.11	0.93-1.33	0.250
Age (Yeas)	1.02	1.01-1.02	<0.001	1.02	1.01-1.02	<0.001
White blood cell (x10^9^/L)	1.01	1.00-1.01	<0.001	1.01	1.00-1.01	0.026
Red blood cell (x10^12^/L)	0.76	0.71-0.81	<0.001	0.85	0.78-0.94	0.001
Hemoglobin (g/L)	0.99	0.99-0.99	<0.001	–	–	–
Platelet (x10^9^/L)	1.00	1.00-1.00	<0.001	1.00	1.00-1.00	<0.001
APTT (s)	0.99	0.98-1.00	0.012	0.95	0.93-0.96	<0.001
Prothrombin Time (s)	1.03	1.01-1.04	<0.001	1.72	1.21-2.45	0.002
INR	1.18	1.05-1.32	0.006	0.01	0.00-0.32	0.008
Fibrinogen (g/L)	1.09	1.05-1.14	<0.001	1.16	1.10-1.23	<0.001
Thrombin Time (s)	1.00	0.98-1.01	0.578	–	–	–
Total bilirubin (μmol/L)	1.00	1.00-1.00	0.001	–	–	–
ALT (IU/L)	1.00	1.00-1.00	0.001	–	–	–
AST (IU/L)	1.00	1.00-1.00	0.011	–	–	–
ALP (IU/L)	1.00	1.00-1.00	<0.001	–	–	–
GGT (IU/L)	1.00	1.00-1.00	<0.001	–	–	–
Total protein (g/L)	0.99	0.99-1.00	0.010	–	–	–
Albumin (g/L)	0.97	0.96-0.98	<0.001	–	–	–
Triglycerides (mmol/L)	1.23	1.18-1.29	<0.001	1.22	1.15-1.30	<0.001
Total Cholesterol (mmol/L)	0.92	0.87-0.96	0.001	–	–	–
HDL Cholesterol (mmol/L)	0.39	0.34-0.46	<0.001	0.54	0.44-0.66	<0.001
LDL Cholesterol (mmol/L)	0.90	0.85-0.96	0.002	–	–	–

ALP, Alkaline Phosphatase; ALT, Alanine Aminotransferase; APTT, Activated Partial Thromboplastin Time; AST, Aspartate Aminotransferase; CI, Confidence Interval; DNA, Deoxyribonucleic Acid; FPG, Fasting Plasma Glucose; GGT, Gamma-glutamyl Transferase; HBeAg, Hepatitis B e Antigen; HBsAg, Hepatitis B Surface Antigen; HBV, Hepatitis B Virus; HCV, Hepatitis C Virus. HDL, High-density Lipoprotein; INR, International Normalized Ratio; LDL, Low-density Lipoprotein; OR, Odds Ratio.

**Table 4:** Univariate and multivariate binary logistic regression analyses exploring potential factors associated with HbA1c ≥6.5%.

Variables	Unadjusted OR	95% CI	p-Value	Adjusted OR	95% CI	p-Value
Sex: Male	1.28	1.02-1.61	0.033	–	–	–
HBsAg: ≥200	1.04	0.84-1.29	0.719	–	–	–
HBeAg: Positive	1.16	0.80-1.67	0.432	–	–	–
HCVAb: Positive	0.94	0.42-2.09	0.874	–	–	–
HBV-DNA	–	–	0.001	–	–	0.003
≥20000 IU/mL	1.66	1.26-2.17	<0.001	1.78	1.31-2.42	<0.001
≥2000, <20000 IU/mL	1.05	0.76-1.45	0.753	1.24	0.87-1.76	0.239
≥100, <2000 IU/mL	0.99	0.76-1.28	0.917	1.17	0.87-1.56	0.300
Age (Yeas)	1.02	1.01-1.03	<0.001	1.02	1.01-1.03	<0.001
White blood cell (x10^9^/L)	1.05	1.02-1.08	0.003	–	–	–
Red blood cell (x10^12^/L)	0.97	0.85-1.10	0.610	–	–	–
Hemoglobin (g/L)	1.00	0.99-1.00	0.144	–	–	–
Platelet (x10^9^/L)	1.00	1.00-1.00	0.178	–	–	–
APTT (s)	0.99	0.97-1.01	0.206	–	–	–
Prothrombin Time (s)	1.02	0.99-1.05	0.226	–	–	–
INR	1.12	0.89-1.41	0.333	–	–	–
Fibrinogen (g/L)	1.16	1.08-1.26	<0.001	1.13	1.04-1.23	0.003
Thrombin Time (s)	1.00	0.99-1.01	0.732	–	–	–
Total bilirubin (μmol/L)	1.00	1.00-1.00	0.318	–	–	–
ALT (IU/L)	1.00	1.00-1.00	0.022	–	–	–
AST (IU/L)	1.00	1.00-1.00	0.212	–	–	–
ALP (IU/L)	1.01	1.01-1.01	<0.001	1.01	1.00-1.01	<0.001
GGT (IU/L)	1.00	1.00-1.00	<0.001	–	–	–
Total protein (g/L)	0.99	0.98-1.01	0.265	–	–	–
Albumin (g/L)	0.97	0.95-0.98	<0.001	–	–	–
Triglycerides (mmol/L)	1.11	1.03-1.19	0.008	1.12	1.03-1.22	0.008
Total Cholesterol (mmol/L)	0.94	0.86-1.02	0.112	–	–	–
HDL Cholesterol (mmol/L)	0.47	0.35-0.63	<0.001	0.65	0.47-0.88	0.006
LDL Cholesterol (mmol/L)	1.02	1.01-1.03	<0.001	–	–	–

ALP, Alkaline Phosphatase; ALT, Alanine Aminotransferase; APTT, Activated Partial Thromboplastin Time; AST, Aspartate Aminotransferase; CI, Confidence Interval; DNA, Deoxyribonucleic Acid; GGT, Gamma-glutamyl Transferase; HbA1c, Glycated Hemoglobin; HBeAg, Hepatitis B e Antigen; HBsAg, Hepatitis B Surface Antigen; HBV, Hepatitis B Virus; HCV, Hepatitis C Virus. HDL, High-density Lipoprotein; INR, International Normalized Ratio; LDL, Low-density Lipoprotein; OR, Odds Ratio.

### 3.2 Correlation of moderately elevated serum HBV-DNA level and type 2 diabetes

The mean age of patients with moderately elevated serum HBV-DNA (≥2000, <20000 IU/mL) was 53.5 (SD: 11.9) years. Among them, there were 1,162 (69.8%) males and 503 (30.2%) females. The patients with total diabetes, FPG ≥7mmol/L and HbA1c ≥6.5% accounted for 14.3% (238/1,665), 9.8% (163/1,665) and 24.4% (65/266), respectively. Univariate binary logistic regression analyses showed that the unadjusted ORs of the correlation of moderately raised serum HBV-DNA level with total diabetes, FPG ≥7mmol/L and HbA1c ≥6.5% relative to negative or lowly elevated serum HBV-DNA (<100 IU/mL) were 0.81 (95% CI: 0.69-0.94, P=0.006), 0.92 (95% CI: 0.76-1.10, P=0.370) and 1.05 (95% CI: 0.76-1.45, P=0.753), respectively. However, multivariate binary logistic regression analyses demonstrated that moderate increase of serum HBV-DNA was not correlated with risk of total diabetes, FPG ≥7mmol/L and HbA1c ≥6.5%, with adjusted ORs of 0.88 (95% CI: 0.72-1.08, P=0.221), 1.00 (95% CI: 0.80-1.25, P=0.993) and 1.24 (95% CI: 0.87-1.76, P=0.239), respectively (**Tables 2**–**4**).

### 3.3 Association of highly increased serum HBV-DNA level and type 2 diabetes

The adult patients with highly elevated serum HBV-DNA level (≥20000 IU/mL) accounted for about one fifth of the total population (2,751/12,527) included in this study. The mean age of this group of people was 51.9 (SD: 13.1) years. Among them, 2,072 patients were males and 679 patients were females. 18.8% (518/2,751) were diabetic. And there were 361 and 123 patients with FPG ≥7mmol/L and HbA1c ≥6.5%, respectively. Compared to patients with negative or lowly elevated serum HBV-DNA (<100 IU/mL), slightly increased and moderately raised serum HBV-DNA, the unadjusted ORs of the relationship of highly raised serum HBV-DNA level with total diabetes were 1.39 (95% CI: 1.18-1.64, P<0.001), 1.13 (95% CI: 0.98-1.29, P=0.084) and 1.12 (95% CI: 1.00-1.26, P=0.060), respectively. **Table 5** showed that the unadjusted ORs of the association of highly raised serum HBV-DNA level with FPG ≥7mmol/L were 1.39 (95% CI: 1.14-1.69, P=0.001), 1.27 (95% CI: 1.08-1.49, P=0.004) and 1.28 (95% CI: 1.11-1.47, P=0.001), respectively. And the unadjusted ORs of the correlation between highly raised serum HBV-DNA level and HbA1c ≥6.5% were 1.57 (95% CI: 1.10-2.24, P=0.012), 1.68 (95% CI: 1.24-2.27, P=0.001) and 1.66 (95% CI: 1.26-2.17, P<0.001), respectively. Moreover, the risk of total diabetes, FPG ≥7mmol/L and HbA1c ≥6.5% in individuals with highly elevated serum HBV-DNA level were 1.38 (95% CI: 1.16-1.65), 1.40 (95% CI: 1.16-1.68) and 1.78 (95% CI: 1.31-2.42) times relative to those with negative or lowly elevated serum HBV-DNA (<100 IU/mL) (**Tables 2**–**4**).

**Table 5:** Binary logistic regression analyses exploring association of different serum levels of HBV-DNA with total diabetes, FPG ≥7mmol/L and HbA1c ≥6.5% (unadjusted OR with 95% CI).

p-value OR (95%CI)	Serum HBV-DNA: ≥ 20000 IU/mL	Serum HBV-DNA: ≥ 2000, <20000 IU/mL	Serum HBV-DNA: ≥ 100, <2000 IU/mL	Serum HBV-DNA: <100 IU/mL
Total diabetes				
Serum HBV-DNA: ≥20000 IU/mL	–	P<0.001 1.39 (1.18-1.64)	P=0.084 1.13 (0.98-1.29)	P=0.060 1.12 (1.00-1.26)
Serum HBV-DNA: ≥2000, <20000 IU/mL	P<0.001 0.72 (0.61-0.85)	–	P=0.015 0.81 (0.69-0.96)	P=0.006 0.81 (0.69-0.94)
Serum HBV-DNA: ≥100, <2000 IU/mL	P=0.084 0.89 (0.77-1.02)	P=0.015 1.23 (1.04-1.46)	–	P=0.921 0.99 (0.88-1.12)
Serum HBV-DNA: <100 IU/mL	P=0.060 0.89 (0.79-1.01)	P=0.006 1.24 (1.06-1.45)	P=0.921 1.01 (0.89-1.14)	–
FPG ≥7mmol/L				
Serum HBV-DNA: ≥20000 IU/mL	–	P=0.001 1.39 (1.14-1.69)	P=0.004 1.27 (1.08-1.49)	P=0.001 1.28 (1.11-1.47)
Serum HBV-DNA: ≥2000, <20000 IU/mL	P=0.001 0.72 (0.59-0.87)	–	P=0.360 0.91 (0.74-1.11)	P=0.370 0.92 (0.76-1.10)
Serum HBV-DNA: ≥100, <2000 IU/mL	P=0.004 0.79 (0.67-0.93)	P=0.360 1.10 (0.90-1.34)	–	P=0.897 1.01 (0.87-1.17)
Serum HBV-DNA: <100 IU/mL	P=0.001 0.78 (0.68-0.90)	P=0.370 1.09 (0.91-1.31)	P=0.897 0.99 (0.85-1.15)	–
HbA1c ≥6.5%				
Serum HBV-DNA: ≥20000 IU/mL	–	P=0.012 1.57 (1.10-2.24)	P=0.001 1.68 (1.24-2.27)	P<0.001 1.66 (1.26-2.17)
Serum HBV-DNA: ≥2000, <20000 IU/mL	P=0.012 0.64 (0.45-0.91)	–	P=0.712 1.07 (0.75-1.51)	P=0.753 1.05 (0.76-1.45)
Serum HBV-DNA: ≥100, <2000 IU/mL	P=0.001 0.60 (0.44-0.80)	P=0.712 0.94 (0.66-1.33)	–	P=0.917 0.99 (0.76-1.28)
Serum HBV-DNA: <100 IU/mL	P<0.001 0.60 (0.46-0.79)	P=0.753 0.95 (0.69-1.31)	P=0.917 1.01 (0.78-1.32)	–

CI, Confidence Interval; DNA, Deoxyribonucleic Acid; FPG, Fasting Plasma Glucose; HbA1c, Glycated Hemoglobin; HBV, Hepatitis B Virus; OR, Odds Ratio.

## 4 Discussion

An epidemiological study has shown that the prevalence of diabetes in Chinese adults is about 12.8% according to the diabetes diagnostic criteria of ADA (28). Meanwhile, it has pointed out that men have a higher risk of diabetes than women. It reported that the prevalence of diabetes in men and women are 13.7% (95% CI: 12.8% to 14.7%) and 11.8% (95% CI: 10.9% to 12.7%), respectively (28). In this study, although the males account for the majority, our results show that the prevalence of total diabetes is around 17.1% in HBsAg-positive adults, which is higher (17.1% VS 13.7%) than that of men alone reported in previous study. Therefore, our results confirms that the prevalence of type 2 diabetes is higher in people with HBV infection. This result has been supported by similar studies in the past. Mehmet Demir et al. (24) found that HBV-DNA was detected in 11% of the diabetic patients (1×10^2^-5×10^3^ copies/ml) and in 3% of the liver health (4×10^3^-1×10^5^ copies/ml). Xu Li et al. (29) found that diabetes mellitus was more prevalent among individuals with HCC (16.1%) compared with those without HCC (7.6%) and increased the risk for HCC by two-fold to three-fold. Studies on gestational diabetes mellitus (GDM) have also been reported. Dongya Wu et al. (23) found that the incidence rates of GDM in the HBV-DNA negative group and HBV-DNA positive group were 18.7% and 19.1%, respectively, significantly higher than that in the control group (p < 0.05). Furthermore, study has reported that the proportion of people with serum HBV-DNA exceeding 20000 IU/mL ranges from 6.4% to 29.2% with an estimated mean value of 10.1% in HBV-infected population (30). And the proportion is highest in southeast Asia and lowest in the Americas. In this study, the percentage of highly increased serum HBV-DNA level (HBV-DNA ≥20000 IU/mL) was approximately 22.0%, which was approximately consistent with that reported in the previous studies.

This study shows that highly elevated serum HBV-DNA level is independently associated with increased risk of total diabetes, increased FPG and HbA1c levels relative to those with negative, mildly or moderately elevated serum HBV-DNA in patients with positive HBsAg. Wu D reports that viral load of HBV-DNA is positively correlated with FPG and HbA1c (23). Although it is a small sample study conducted among pregnant women with HBV infection, its results are consistent with the results of this study.

Based on this, a deeper understanding of the relationship between type 2 diabetes and HBV infection is necessary. Bingfeng Han et al. (31) used two stratified analyses to explore the relationship between diabetes and HBV infection. These two results indicated that HBV infection may be related directly to self-monitoring of blood glucose (SMBG) at home, not diabetes. Interestingly, Nicola D Thompson et al. (32) reported that the people with diabetes have shown that HBV infection, especially acute HBV infection, was probably transmitted during SMBG. This means that HBV infection can be caused by parenteral contamination in patients auto-monitoring for diabetes. Jian Zhang et al. (33) reported a meta-analysis which suggested that while HBV itself may not be pro-diabetic, the HBV-derived cirrhosis is an independent risk factor for type 2 diabetes. Moreover, B F Han et al. (34) found that patients with diabetes are more likely to be infected with HBV than those without diabetes, Diabetes mellitus is likely to be a risk factor for HBV infection. Briefly, interventions on high-risk population is essential to reduce hepatitis B incidence, and patients with diabetes need to be counted.

There may be some underlying mechanisms of the association between high serum HBV-DNA level and increased risk of type 2 diabetes. Large quantity of replicated HBV in host will cause inflammatory necrosis of hepatocytes resulting in the reduced inactivation of insulin and glucagon, which will lead to impaired glucose tolerance and insulin resistance (35–37). On the other hand, the synthesis of hexokinase and glycogen synthase in the liver slows down after hepatocytes damage (38, 39). The decreased activity of hexokinase and glycogen synthase can affect the uptake and utilization of glucose resulting in the increase of blood glucose. It is found that HBV-DNA or HBsAg exists not only in hepatocytes, but also in pancreatic tissue and pure pancreatic juice (40–42). Studies report that HBV replication in the pancreas leads to pancreatic autoimmune response (37, 41). This suggests that HBV can directly or through immune-mediated to damage the β cells in pancreatic islets (35, 42, 43). This may be the direct cause of diabetes in HBV-infected people with highly increased serum HBV-DNA. In addition, some researchers believe that interferon, as a first-line drug for the treatment of HBV, will cause or further aggravate the increase of blood glucose or induce the injury of pancreatic islet cells in some patients with HBV infection (44–47).

This study has several limitations. First, this was a cross-sectional study from a single center. Second, some potential confounding factors which had not been selected into analyses may affect the results. Third, among 54,219 cases obtained from the database system of our hospital, about three-quarters of subjects were excluded for lacking of the results of HBV-DNA test and only 12,527 patients were tested for serum HBV-DNA. Somewhat the prevalence of type 2 diabetes reported in this study may be overestimated. As another limitation, this study was unable to identify a link between HBV infection and type 2 diabetes. Therefore, whether HBV infection triggers the onset of type 2 diabetes or type 2 diabetes increases the risk of HBV infection remains to be determined by prospective or longitudinal studies. Considering the complications associated with the coexistence of hepatitis B and type 2 diabetes, vaccination and screening of all diabetic patients for HBV infection as well as timely treatment of infected patients are recommended to reduce the incidence and risk of HBV infection among the diabetic population. In addition, in this study the minimum value of quantitative detection of serum HBV-DNA was 100 IU/mL, so patients with negative or lowly increased serum HBV-DNA cannot be identified and were classified into one group. Finally, because only the patients who reported having type 2 diabetes by oneself or with abnormal FPG had tested blood HbA1c, a proportion of the included people had no results of HbA1c. Although the data of this study was based on the data of hospital inpatients, we believed the results of the analyses of large sample were convincing.

In conclusion, our findings indicate that slightly to moderately elevated serum HBV-DNA levels are not associated with the risk of type 2 diabetes in patients with positive HBsAg. However, the extremely high serum HBV-DNA level may increase the risk of total diabetes, FPG ≥7mmol/L and HbA1c ≥6.5%. Therefore, we speculate that controlling serum HBV-DNA levels may reduce the risk of type 2 diabetes in HBV-infected patients. We look forward to multicenter and prospective studies with large sample to confirm the results. And the experimental researches are needed to reveal the potential mechanisms.

## Data availability statement

The original contributions presented in the study are included in the article/supplementary material. Further inquiries can be directed to the corresponding author.

## Ethics statement

The studies involving human participants were reviewed and approved by Medical Ethics Committee of Tongji Medical College, Huazhong University of Science and Technology. The patients/participants provided their written informed consent to participate in this study.

## Author contributions

SZ and YZ contributed equally to this work. SZ, YZ and GX had full access to the data in the study and take responsibility for the integrity of the data and the accuracy of the data analyses. All authors were responsible for the study concept and design. SZ, YZ and GX were responsible for the acquisition and analyses of data. All authors contributed to the article and approved the submitted version.
